# Consistent sleep onset and maintenance of body weight after weight loss: An analysis of data from the NoHoW trial

**DOI:** 10.1371/journal.pmed.1003168

**Published:** 2020-07-16

**Authors:** Sofus C. Larsen, Graham Horgan, Marie-Louise K. Mikkelsen, Antonio L. Palmeira, Sarah Scott, Cristiana Duarte, Inês Santos, Jorge Encantado, Ruairi O'Driscoll, Jake Turicchi, Joanna Michalowska, R. James Stubbs, Berit L. Heitmann

**Affiliations:** 1 Research Unit for Dietary Studies, The Parker Institute, Bispebjerg and Frederiksberg Hospital, The Capital Region, Denmark; 2 Biomathematics and Statistics Scotland, Aberdeen, United Kingdom; 3 Centro Interdisciplinar para o Estudo da Performance Humana, Faculdade de Motricidade Humana, Universidade de Lisboa, Lisbon, Portugal; 4 School of Psychology, Faculty of Medicine and Health, University of Leeds, Leeds, United Kingdom; 5 Laboratório de Nutrição, Faculdade de Medicina, Universidade de Lisboa, Lisbon, Portugal; 6 The Boden Institute of Obesity, Nutrition, Exercise & Eating Disorders, The University of Sydney, Sydney, Australia; 7 Department of Public Health, Section for General Practice, University of Copenhagen, Copenhagen, Denmark; Uppsala Universitet, SWEDEN

## Abstract

**Background:**

Several studies have suggested that reduced sleep duration and quality are associated with an increased risk of obesity and related metabolic disorders, but the role of sleep in long-term weight loss maintenance (WLM) has not been thoroughly explored using prospective data.

**Methods and findings:**

The present study is an ancillary study based on data collected on participants from the Navigating to a Healthy Weight (NoHoW) trial, for which the aim was to test the efficacy of an evidence-based digital toolkit, targeting self-regulation, motivation, and emotion regulation, on WLM among 1,627 British, Danish, and Portuguese adults. Before enrolment, participants had achieved a weight loss of ≥5% and had a BMI of ≥25 kg/m^2^ prior to losing weight. Participants were enrolled between March 2017 and March 2018 and followed during the subsequent 12-month period for change in weight (primary trial outcome), body composition, metabolic markers, diet, physical activity, sleep, and psychological mediators/moderators of WLM (secondary trial outcomes). For the present study, a total of 967 NoHoW participants were included, of which 69.6% were women, the mean age was 45.8 years (SD 11.5), the mean baseline BMI was 29.5 kg/m^2^ (SD 5.1), and the mean weight loss prior to baseline assessments was 11.4 kg (SD 6.4). Objectively measured sleep was collected using the Fitbit Charge 2 (FC2), from which sleep duration, sleep duration variability, sleep onset, and sleep onset variability were assessed across 14 days close to baseline examinations. The primary outcomes were 12-month changes in body weight (BW) and body fat percentage (BF%). The secondary outcomes were 12-month changes in obesity-related metabolic markers (blood pressure, low- and high-density lipoproteins [LDL and HDL], triglycerides [TGs], and glycated haemoglobin [HbA1c]). Analysis of covariance and multivariate linear regressions were conducted with sleep-related variables as explanatory and subsequent changes in BW, BF%, and metabolic markers as response variables. We found no evidence that sleep duration, sleep duration variability, or sleep onset were associated with 12-month weight regain or change in BF%. A higher between-day variability in sleep onset, assessed using the standard deviation across all nights recorded, was associated with weight regain (0.55 kg per hour [95% CI 0.10 to 0.99]; *P* = 0.016) and an increase in BF% (0.41% per hour [95% CI 0.04 to 0.78]; *P* = 0.031). Analyses of the secondary outcomes showed that a higher between-day variability in sleep duration was associated with an increase in HbA1c (0.02% per hour [95% CI 0.00 to 0.05]; *P* = 0.045). Participants with a sleep onset between 19:00 and 22:00 had the greatest reduction in diastolic blood pressure (DBP) (*P* = 0.02) but also the most pronounced increase in TGs (*P* = 0.03). The main limitation of this study is the observational design. Hence, the observed associations do not necessarily reflect causal effects.

**Conclusion:**

Our results suggest that maintaining a consistent sleep onset is associated with improved WLM and body composition. Sleep onset and variability in sleep duration may be associated with subsequent change in different obesity-related metabolic markers, but due to multiple-testing, the secondary exploratory outcomes should be interpreted cautiously.

**Trial registration:**

The trial was registered with the ISRCTN registry (ISRCTN88405328).

## Abstract 2

Sofus Larsen and colleagues reveal the importance of consistent sleep patterns to avoid weight gain after weight loss

## Author summary

**Why was this study done?:**

- Weight loss maintenance (WLM) is the greatest challenge in successful treatment of obesity.
- While several studies have suggested that reduced sleep duration and quality are associated with an increased risk of obesity, the role of sleep in long-term WLM has not been thoroughly examined.

**What did the researchers do and find?:**

- This study included a total of 967 men and women who had achieved a clinically significant weight loss (≥5%) during the last 12 months and had a BMI of ≥25 kg/m^2^ prior to their weight loss.
- Sleep duration, sleep duration variability, sleep onset, and sleep onset variability were assessed across 14 days close to baseline examinations using the Fitbit Charge 2 (FC2) device, and changes in body weight (BW), composition, and obesity-related metabolic markers were followed up after 12 months.
- While no associations were found between sleep duration, sleep duration variability, or sleep onset and subsequent WLM, participants with a more variable sleep onset at baseline regained more weight and had a higher increase in body fat percentage (BF%) during the 12-month follow-up period.

**What do these findings mean?:**

- These results suggest that maintaining a consistent sleep onset may be associated with improved WLM and body composition.
- Due to the observational design, the study cannot confirm whether this relationship is causal or a reflection of different personality traits related to sleep onset variability and energy balance behaviours.

## Introduction

Several studies have found that obesity is strongly associated with both morbidity and mortality [1,2], which has raised serious public health concerns, as the prevalence of obesity is increasing on a global level [3]. The failure to combat the obesity epidemic is not due to the lack of effective weight loss methods. Many existing approaches are effective for initial weight loss [4], but behavioural interventions have generally shown limited effects on long-term weight loss maintenance (WLM) [5]. The lack of effective interventions may suggest a need to identify early prognostic markers related to successful WLM, and while many studies have already been conducted, they have largely focused on diet and physical activity [6].

The evidence is sparse, but recently published data suggest that self-reported sleep duration and quality may be associated with improved WLM [7–9], and several studies have suggested potential mechanisms by which insufficient sleep could hinder successful WLM, including metabolic changes affecting glucose metabolism, appetite and reward, caloric intake, and/or energy expenditure [10]. Whereas few studies have specifically focused on WLM, the evidence linking short sleep duration to weight gain, obesity, and related metabolic disorders has increased substantially during the last decades [11–16]. Not only the duration of sleep but also sleep quality parameters, such as timing and variability in sleep, have been suggested as potential causes of obesity [17]. Moreover, a recently published study from the PREDIMED Plus lifestyle intervention suggested that low variability in sleep duration in addition to an adequate average sleep duration was associated with improved 12-month weight loss in individuals with obesity [18].

The present study is an ancillary study based on data collected on participants from the Navigating to a Healthy Weight (NoHoW) trial, in which the aim was to test the efficacy of an evidence-based digital toolkit, targeting self-regulation, motivation, and emotion regulation, on WLM among British, Danish, and Portuguese adults. Using the NoHoW data, the primary aims of the present study were to examine if sleep duration and sleep patterns were associated with subsequent 12-month change in body weight (ΔBW) and body fat percentage (ΔBF%). Secondarily, 12-month changes in obesity-related metabolic markers (including systolic blood pressure [ΔSBP], diastolic blood pressure [ΔDBP], low-density lipoproteins [ΔLDL], high-density lipoprotein [ΔHDL], triglycerides [ΔTG], and glycated haemoglobin [ΔHbA1c]) were examined as exploratory outcomes.

## Methods and materials

This study is reported according to the Strengthening the Reporting of Observational Studies in Epidemiology (STROBE) statement (S1 STROBE Checklist). An analysis plan covering the primary outcomes was developed prior to starting any statistical analysis (S1 Text). However, the secondary exploratory outcomes were not part of the analysis plan.

### Study population

A detailed description of the NoHoW trial can be found elsewhere [19]. In brief, the NoHoW trial was a randomised controlled trial testing the efficacy of an information and communications technology (ICT)-based toolkit to support WLM in the United Kingdom (Leeds), Denmark (Copenhagen), and Portugal (Lisbon). At baseline examinations between March 2017 and March 2018, all participants received a Fitbit Charge 2 (FC2; San Francisco, CA) device, which collected data on sleep and physical activity, and were randomly allocated to one of four arms: (1) self-monitoring only (self-weighing and activity tracker), (2) self-regulation plus motivation, (3) emotion regulation, or (4) combined self-regulation, motivation, and emotion regulation. Participants were randomly allocated to treatment arms by researchers using an online trial administration portal. Adaptive stratified sampling using minimisation was embedded in the portal using the R-programme (R Foundation for Statistical Computing, Vienna, Austria). This minimised the differences in age, weight loss in the 12 months prior to enrolment, and baseline BMI between treatment arms and stratified by sex and country. Participants were blinded to study allocation. The research team was not blinded due to the need to train participants in arm-specific toolkit versions. Participants were followed during a 12-month period for change in weight (primary trial outcome), body composition, metabolic markers, dietary intake, physical activity, sleep, and psychological mediators/moderators of WLM (secondary trial outcomes). Participants were 18 years or older, had achieved a verified and clinically significant weight loss (≥5%) within the previous 12 months, and had a BMI (prior to weight loss) of ≥25 kg/m^2^. The exclusion criteria were as follows: achieved weight loss due to illness or surgical procedures; were pregnant or breastfeeding; involved in other research intervention studies that confound with the aims of the intervention; unable to follow written material or telephone conversations in the English, Danish, or Portuguese language (depending on the trial centre); diagnosed with an eating disorder; diagnosed with any condition that may interfere with increasing mild to moderate physical activities and that is unstable (i.e., untreated or unable to be controlled by medication); recently diagnosed with type 1 diabetes; planning extensive travel (e.g., more than four weeks); or living in the same household as existing participant in the trial [19].

A total of 1,627 participants were enrolled in the NoHoW trial. The present ancillary study was based on information collected at baseline and 12-month follow-up visits. For this study, we further excluded participants with missing or insufficient data on objectively measured sleep or physical activity (<7 days with information on a main sleep periods as defined by the FC2 [*n* = 229], mean sleep onset between 06:00 and 19:00 [*n* = 3], and <6 valid days and 2 weekend days of physical activity [*n* = 115]), missing information on ΔBW or ΔBF% (*n* = 266) and selected baseline covariates (*n* = 47). This resulted in a total of 967 participants in the final analyses of primary outcomes and a slightly lower sample size in analyses of secondary outcomes (SPB [*n* = 965], DBP [*n* = 965], LDL [*n* = 880], HDL [*n* = 913], TG [*n* = 922], and HbA1c [*n* = 954]). A flowchart showing the selection of participants can be found in **S1 Fig.**

### Ethics

The study was conducted in accordance with the Helsinki Declaration. Ethical approval was granted by local institutional ethics committees at the Universities of Leeds (17–0082; 27 February 2017), Lisbon (17/2016; 20 February 2017) and the Capital Region of Denmark (H-16030495; 8 March 2017).

### FC2

Sleep duration and physical activity were estimated using the FC2, a wrist-worn activity tracker with a triaxial accelerometer, providing information on physical activity, sleep, and heart rate [20]. According to the provider, the device estimates sleep from equations using information from a combination of movement and heart rate patterns [21]. The device has been validated against polysomnography [22], but the exact algorithms used by Fitbit have not been disclosed.

At the baseline visits between March 2017 and March 2018, a Fitbit account was created for each participant, and the Fitbit app was downloaded to their personal phone, tablet, or computer. Information on age, sex, height, and weight were added to all accounts. The device was updated to the latest firmware and placed on the nondominant wrist of the participant. Participants were instructed to wear the FC2 for the duration of the study, apart from during water activities (e.g., showering) and charging the device. In the present study, we used 14 days of sleep data recorded from day 3 to day 16 of the intervention. Days 1 and 2 were excluded to make sure all devices had been properly set up. The limitation to two weeks of data was chosen as the compromise between getting a sufficient number of data to represent a robust measure of habitual sleep and still being able to consider the sleep variables’ baseline measures.

From this information, mean total sleep duration was calculated and included in analyses as a categorical variable: <6, 6–<7, 7–<8, 8–<9, and ≥9 hours [23]. Moreover, mean sleep onset was assessed by identifying the beginning of each main sleep period in minutes from midnight (e.g., 23:00 = −60 minutes and 01:00 = 60 minutes). For each individual, daily sleep onset was then averaged across all nights to obtain an estimate of mean sleep onset, which was included in the following time categories: 19:00–<22:00, 22:00–<00:00, 00:00–<03:00, and 03:00–<06:00. Variability in sleep duration and sleep onset was estimated for each individual using the standard deviation across all nights recorded and included as a continuous variable (hours).

When no heart rate data were available from the FC2, we considered it as non-wear time. To avoid loss of data due to connectivity issues, gaps of less than 10 minutes were imputed with the average of the last measured and the next observed heart rate. Minute-level data were aggregated to hourly data and missing time was determined per hour. Total steps were divided by the number of minutes the device was worn, on the assumption that data missing within each hour were most representative of missing data. Hours with more than 30 minutes of missing data were removed from the data. Next, hourly averages were summed per day, a minimum of 21 valid hours were required for a valid day. Lastly, total steps were averaged across the 14-day period if at least 6 valid days and 2 weekend days were available. From this, physical activity measured as average steps/day (continuous variable) was included as a potential confounder.

### Anthropometry and biomarkers

At baseline and after the 12 months of follow-up, BW was measured to the nearest 0.1 kg and height was measured at baseline to the nearest 0.1 cm using the Seca 704s (SECA, Hamburg, Germany) combined stadiometer and electronic scale. Body composition was estimated using bioelectrical impedance using the ImpediMed SFB7 device (ImpediMed, Inc, Sydney, Australia) (software version 5.4.0), following the manufacturer’s instructions. We used the Moissl BMI modification of the mixture theory equations to determine BF%, a method that has been found appropriate over a wide range of body compositions [24]. Blood pressure was measured in a sitting position using the automatic sphygmomanometer Microlife BP A2 (Microlife, Taipei, Taiwan). Blood lipids and HbA1c were measured in fasted state (approximately 10 hours) by a finger-prick test using the point-of-care instrument Alere Afinion AS100 analyser (Abbott Laboratories, Abbott Park, IL) [19,25,26]. Changes in primary and secondary outcome measures (follow-up values minus baseline values) were included in analyses as continuous variables (ΔBW [kg per year], ΔBF% [% per year], ΔSBP [mm Hg per year], ΔDBP [mm Hg per year], ΔLDL [mmol/L per year], ΔHDL [mmol/L per year], ΔTG [mmol/L per year], and ΔHbA1c [% per year]).

### Covariates

All potential confounding factors were selected a priori based on previous research [12], biological plausibility, and available information. Participants provided verified information on weight loss during the 12 months prior to baseline (by a health professional, WL counsellor/friend, WL programme record booklet, diary, smartphone app, or before/after photographs), and these data were included in the analyses as a continuous variable (kg). At baseline, all participants were allocated to an intervention arm and this information was included as a categorical variable: (1) self-monitoring only (self-weighing and activity tracker), (2) self-regulation plus motivation, (3) emotion regulation, or (4) combined self-regulation plus motivation and emotion regulation. Participants were asked to report their smoking status in one of the following categories: current smoker, previous smoker (quit in last 5 years), previous smoker (quit more than 5 years ago), or never smoked. For the present study, previous smoker (quit in last 5 years) and previous smoker (quit more than 5 years ago) were collapsed into one category. Likewise, information was collected on alcohol consumption: ‘During the last 12 months, how often did you usually have any kind of drink containing alcohol? By a drink we mean a unit (every day, 5–6 times a week, 3–4 times a week, twice a week, once a week, 2–3 times a month, 3–11 times in the past year, 1 or 2 times in the past year, I did not drink any alcohol in the past year, but I did drink in the past, or I never drank alcohol in my life)’. In the present study, alcohol consumption was included using the following 6 categories: every day, 5–6 times a week, 3–4 times a week, twice a week, once a week, and less than once a week. Information on highest level of education was provided and categorised according to the International Standard Classification of Education (ISCED) [27] as high, medium, low, or other (including educations not classified by ISCED). Psychosocial stress was assessed with the short version of the perceived stress scale [28,29]. This scale has the following four items that focus on the assessment of stress and coping over the preceding month: ‘How often have you felt that you were unable to control the important things in your life?’ ‘How often have you felt confident about your ability to handle your personal problems?’ ‘How often have you felt that things were going your way?’ and ‘How often have you felt difficulties were piling up so high that you could not overcome them?’ Responses were made on a 5-point Likert scale (never, rarely, sometimes, often, and very often). The items were then summed to give a total perceived stress score with a range of 0 (least stressed) to 16 (most stressed), which was included as a continuous variable. Finally, information on age (continuous variable) and sex was included.

### Statistical analyses

The sample size of the NoHoW trial was established with the main purpose of having enough statistical power to detect a potential effect of the intervention on the primary outcome (ΔBW) [19]. As the present results represent an ancillary study, the sample size was not determined specifically for the purpose of these analyses. However, the pre-established sample size of 967 individuals gave approximately 85% power to detect correlations with absolute values of 0.1 or greater.

Analysis of covariance was used to test differences in outcome measures (ΔBW, ΔBF%, ΔSBP, ΔDBP, ΔLDL, ΔHDL, ΔTG, and ΔHbA1c) across categories of sleep duration (<6, 6–<7, 7–<8, 8–<9, and ≥9 hours) and sleep onset (19:00–<22:00, 22:00–<00:00, 00:00–<03:00, and 03:00–<06:00). These results were presented as estimated marginal means in categories of sleep duration and sleep onset. Multivariate linear regression was used to examine the association between variability in sleep duration or sleep onset and subsequent change in outcome measures, presented as change in outcomes per additional hour of between-day variability in baseline sleep duration or sleep onset. First, crude models, including information on outcome, exposure, and baseline measure of outcome only, were conducted. Secondly, adjusted analyses with added information on initial weight loss, smoking status, frequency of alcohol consumption, physical activity, education, perceived stress, age, intervention status (arm allocation), and sex as potential confounding factors were carried out. To get associations independent of total sleep duration, analyses of sleep duration variability, sleep onset, and sleep onset variability were additionally adjusted for baseline sleep duration in a third model.

Sex and intervention interactions were tested in all analyses of primary outcomes by adding product terms to the models, and subgroup analyses were conducted if appropriate. Although not optimal, as this limits comparability with prior analysis, for this purpose, the five item sleep duration variable was collapsed into four categories (<7, 7–8, 8–9, and ≥9 hours) and the four-item sleep onset variable was collapsed into three categories (19:00–22:00, 22:00–00:00, 00:00–06:00) due to few individuals with a sleep duration <6 hours (*n* = 23) and a sleep onset between 03:00 and 06:00 (*n* = 20).

Normality of continuous variables and model assumptions (investigating linearity of effects on outcomes, consistency with a normal distribution, and variance homogeneity) were assessed through visual inspection of histograms and residual plots.

All statistical tests were two-tailed, with a significance level at 0.05. Analyses were performed using Stata SE 14 (StataCorp LP, College Station, TX). All figures were produced with SigmaPlot 13.0 (San Jose, CA).

### Sensitivity analyses

In order to capture potentially nonlinear associations, we included sleep duration and sleep onset as categorical variables. However, to maximise statistical power, we also performed analyses of primary outcomes, with sleep duration and sleep onset included as continuous variables. Moreover, although most of the included individuals had information on sleep duration from a relatively large proportion of the 14-day baseline period, some individuals only had 7 days of data available. Thus, for the primary outcomes, sensitivity analyses were also conducted, adjusting for the number of available days.

## Results

A total of 294 men and 673 women were included in study. A higher mean age was observed among women (46.6 years [SD: 11.9]) than men (43.8 years [SD 10.4]; *P* < 0.001). The mean BMI was 29.5 (SD 5.1) with no statistically significant difference between the sexes (*P* = 0.957) **(Table 1).**

**Table 1:** Baseline characteristics of men and women from the NoHoW study.

Characteristics	All (*n* = 967)^1^	Men (*n* = 294)	Women (*n* = 673)	*P* value^2^
Age (years)	45.8 (11.5)	43.8 (10.4)	46.6 (11.9)	<0.001
Height (cm)	168.9 (8.3)	177.0 (6.4)	165.5 (6.6)	<0.001
BMI (kg/m2)	29.5 (5.1)	29.4 (4.5)	29.6 (5.3)	0.957
Initial weight loss (kg)	−11.4 (6.4)	−11.5 (6.9)	−11.4 (6.2)	0.992
PSS (possible range: 0–16)	5.8 (2.8)	5.4 (2.7)	5.9 (2.9)	0.005
Frequency of alcohol consumption (%)				
Every day	2.0	3.4	1.3	<0.001
5–6 times a week	3.4	5.4	2.5	
3–4 times a week	11.5	16.0	9.5	
Twice a week	15.7	19.1	14.3	
Once a week	10.8	13.3	9.7	
<once a week	56.7	42.9	62.7	
Smoking status (%)				
Current	6.9	8.8	6.1	0.289
Previous	40.6	40.5	40.7	
Never	52.4	50.7	53.2	
Educational status (%)				
Low	9.1	7.1	10.0	0.020
Medium	20.5	26.2	18.0	
High	66.1	61.9	67.9	
Other	4.3	4.8	4.2	
Intervention group (%)				
Control	25.2	23.8	25.9	0.873
Self-regulation and motivation	23.9	23.5	24.1	
Stress and emotion regulation	25.2	26.5	24.7	
Stress and emotion regulation + self-regulation and motivation	25.7	26.2	25.4	

^1^Results presented as mean (SD) unless otherwise stated.

^2^*P* value for sex difference (Wilcoxon rank-sum test or chi-squared test).

Abbreviations: NoHoW, Navigating to a Healthy Weight; PSS, perceived stress scale

Sleep duration and quality was collected during an average of 12.0 days (SD 1.7) throughout the 14-day baseline period. The mean sleep duration was 7.8 hours (SD 0.9), and a higher duration of sleep was observed among women (8.0 hours [SD 0.8]) than men (7.4 hours [SD 1.0]; *P* < 0.001). The variability in sleep duration, expressed as the standard deviations across all nights, was 1.4 hours (SD 0.7), with identical estimates for men and women. The mean sleep onset was 11 minutes to midnight (SD 74), and a later sleep onset was observed among men (23 minutes past midnight [SD 83]) than women (26 minutes to midnight [SD 64]; *P* < 0.001). The variability in sleep onset, expressed as the standard deviation across all nights, was 1.3 hours (SD: 0.9), with a slightly higher variability observed among men (1.4 hours [SD 1.0]) than women (1.3 [SD 0.8]; *P* = 0.014) (**Table 2**).

**Table 2:** Information on Fitbit data, adiposity, and health markers among men and women from the NoHoW study.

Parameters	All (*n* = 967)^1^	Men (*n* = 294)	Women (*n* = 673)	*P* value^2^
**Fitbit data**				
Sleep duration (hours/day)	7.8 (0.9)	7.4 (1.0)	8.0 (0.8)	<0.001
Variation daily sleep duration (hours)	1.4 (0.7)	1.4 (0.6)	1.4 (0.8)	0.444
Sleep onset (minutes from midnight)	−11 (74)	23 (83)	−26 (64)	<0.001
Variation in sleep onset (hours)	1.3 (0.9)	1.4 (1.0)	1.3 (0.8)	0.014
Sleep (number of days available)	12.0 (1.7)	12.0 (1.8)	12.0 (1.7)	0.985
Physical activity (steps/day)	10,756 (3,411)	11,599 (3,692)	10,388 (3,215)	<0.001
**Primary outcomes**				
Baseline BW (kg)	84.4 (16.5)	91.8 (16.1)	81.2 (15.7)	<0.001
ΔBW (kg)	0.2 (6.2)	−0.1 (5.8)	0.3 (6.3)	0.162
Baseline BF%	37.8 (9.1)	31.2 (8.0)	40.7 (7.9)	<0.001
ΔBF%	−0.7 (5.5)	−0.9 (5.2)	−0.6 (5.6)	0.361
**Secondary outcomes**^**3**^				
Baseline SBP (mm Hg)	121.9 (14.8)	127.2 (13.7)	119.6 (14.7)	<0.001
ΔSBP (mm Hg)	−1.4 (10.7)	−2.8 (10.1)	−0.7 (10.9)	0.008
Baseline DBP (mm Hg)	76.2 (9.1)	79.9 (8.9)	74.6 (8.7)	<0.001
ΔDBP (mm Hg)	−0.2 (6.9)	−0.9 (6.9)	0.4 (6.9)	0.072
Baseline LDL (mmol/L)	2.8 (0.9)	2.8 (0.8)	2.8 (0.9)	0.177
ΔLDL (mmol/L)	−0.0 (0.7)	0.0 (0.7)	−0.1 (0.7)	0.061
Baseline HDL (mmol/L)	1.6 (0.4)	1.4 (0.3)	1.7 (0.4)	<0.001
ΔHDL (mmol/L)	0.2 (0.3)	0.1 (0.3)	0.2 (0.3)	0.155
Baseline TG (mmol/L)	1.3 (0.9)	1.3 (0.9)	1.3 (0.8)	0.973
ΔTG (mmol/L)	0.3 (1.1)	0.2 (1.1)	0.3 (1.1)	0.166
Baseline HbA1c (%)	5.2 (0.5)	5.2 (0.6)	5.2 (0.4)	0.004
ΔHbA1c (%)	0.1 (0.3)	0.1 (0.3)	0.1 (0.2)	0.439

^1^Results presented as mean (SD) unless otherwise stated.

^2^*P* value for sex difference (Wilcoxon rank-sum test or chi-squared test).

^3^Reduced sample size for secondary outcomes: SPB (*N* = 965), DBP (*N* = 965), LDL (*N* = 880), HDL (*N* = 913), TG (*N* = 922), HbA1c (*N* = 954).

Abbreviations: BF%, body fat percentage; BW, body weight; DBP, diastolic blood pressure; HbA1c, glycated haemoglobin; HDL, high-density lipoprotein; LDL, low-density lipoprotein; NoHoW, Navigating to a Healthy Weight; SBP, systolic blood pressure; TG, triglyceride; Δ, 12-month change

### Sleep duration and sleep onset

After adjusting for potential confounders, no associations between baseline sleep duration and ΔBW, ΔBF%, or change in any of the secondary outcomes were observed **(Table 3)**.

**Table 3:** Mean values of 12-month changes in BW, composition, and secondary outcomes in categories of baseline sleep duration.

	<6 hours (*n* = 23)		6–7 hours (*n* = 153)		7–8 hours (*n* = 399)		8–9 hours (*n* = 317)		≥9 hours (*n* = 75)		
	Mean^1^	(95% CI)	Mean	(95% CI)	Mean	(95% CI)	Mean	(95% CI)	Mean	(95% CI)	*P*^4^
**ΔBW (kg)**	** **	** **			** **	** **			** **	** **	
Crude^2^	−1.96	(−4.48 to 0.57)	0.15	(−0.82 to 1.12)	0.36	(−0.24 to 0.96)	0.20	(−0.48 to 0.87)	−0.28	(−1.67 to 1.10)	0.471
Adjusted^3^	−1.89	(−4.47 to 0.70)	0.20	(−0.79 to 1.19)	0.34	(−0.25 to 0.94)	0.22	(−0.46 to 0.90)	−0.42	(−1.81 to 0.98)	0.465
**ΔBF%**											
Crude	−2.31	(−4.46 to −0.17)	−0.95	(−1.79 to −0.12)	−0.78	(−1.30 to −0.27)	−0.46	(−1.04 to 0.12)	−0.26	(−1.45 to 0.93)	0.437
Adjusted	−1.00	(−3.16 to 1.16)	−0.64	(−1.47 to 0.19)	−0.80	(−1.30 to −0.30)	−0.56	(−1.14 to 0.01)	−0.76	(−1.93 to 0.41)	0.976
**ΔSBP (mm Hg)**											
Crude	−2.32	(−6.36 to 1.72)	−2.07	(−3.63 to −0.51)	−0.74	(−1.70 to 0.22)	−1.86	(−2.95 to −0.77)	−0.72	(−2.94 to 1.50)	0.441
Adjusted	−2.68	(−6.78 to 1.43)	−2.29	(−3.86 to −0.71)	−0.85	(−1.80 to 0.10)	−1.61	(−2.70 to −0.52)	−0.62	(−2.85 to 1.61)	0.490
**ΔDBP (mm Hg)**											
Crude	−0.60	(−3.27 to 2.08)	0.25	(−0.78 to 1.28)	−0.25	(−0.89 to 0.39)	−0.48	(−1.20 to 0.24)	0.00	(−1.47 to 1.48)	0.832
Adjusted	−0.73	(−3.48 to 2.02)	0.13	(−0.92 to 1.19)	−0.29	(−0.93 to 0.34)	−0.36	(−1.09 to 0.37)	−0.02	(−1.52 to 1.47)	0.932
**ΔLDL (mmol/L)**											
Crude	0.21	(−0.06 to 0.48)	−0.03	(−0.14 to 0.07)	−0.02	(−0.08 to 0.05)	−0.07	(−0.14 to 0.00)	−0.03	(−0.18 to 0.12)	0.337
Adjusted	0.12	(−0.16 to 0.40)	−0.06	(−0.17 to 0.04)	−0.02	(−0.08 to 0.05)	−0.05	(−0.13 to 0.02)	−0.01	(−0.16 to 0.14)	0.695
**ΔHDL (mmol/L)**											
Crude	0.13	(0.01 to 0.25)	0.17	(0.12 to 0.22)	0.16	(0.14 to 0.19)	0.14	(0.11 to 0.18)	0.18	(0.11 to 0.24)	0.795
Adjusted	0.18	(0.06 to 0.30)	0.18	(0.13 to 0.22)	0.17	(0.14 to 0.20)	0.14	(0.10 to 0.17)	0.15	(0.08 to 0.22)	0.561
**ΔTG (mmol/L)**											
Crude	−0.02	(−0.46 to 0.41)	0.29	(0.12 to 0.46)	0.27	(0.17 to 0.38)	0.33	(0.21 to 0.44)	0.36	(0.12 to 0.60)	0.595
Adjusted	−0.01	(−0.46 to 0.44)	0.28	(0.11 to 0.45)	0.26	(0.16 to 0.37)	0.34	(0.22 to 0.46)	0.37	(0.12 to 0.62)	0.571
**ΔHbA1c (%)**											
Crude	0.11	(0.02 to 0.21)	0.08	(0.04 to 0.12)	0.07	(0.05 to 0.09)	0.09	(0.07 to 0.12)	0.06	(0.01 to 0.12)	0.668
Adjusted	0.08	(-0.02 to 0.18)	0.07	(0.04 to 0.11)	0.07	(0.04 to 0.09)	0.10	(0.07 to 0.13)	0.07	(0.02 to 0.13)	0.440

^1^Results presented as mean 12-month change (95% CI) in categories of sleep duration.

^2^Model with information on outcome, exposure, and baseline measure of outcome.

^3^Adjusted for intervention status, initial weight loss, physical activity, perceived stress, smoking status, frequency of alcohol consumption, education, sex, and age.

^4^*P* values for difference between sleep duration categories were produced using analysis of covariance.

Abbreviations: BF%, body fat percentage; BW, body weight; DBP, diastolic blood pressure; HbA1c, glycated haemoglobin; HDL, high-density lipoprotein; LDL low-density lipoprotein; SBP, systolic blood pressure; TG, triglyceride; Δ, 12-month change

**Fig 1:**
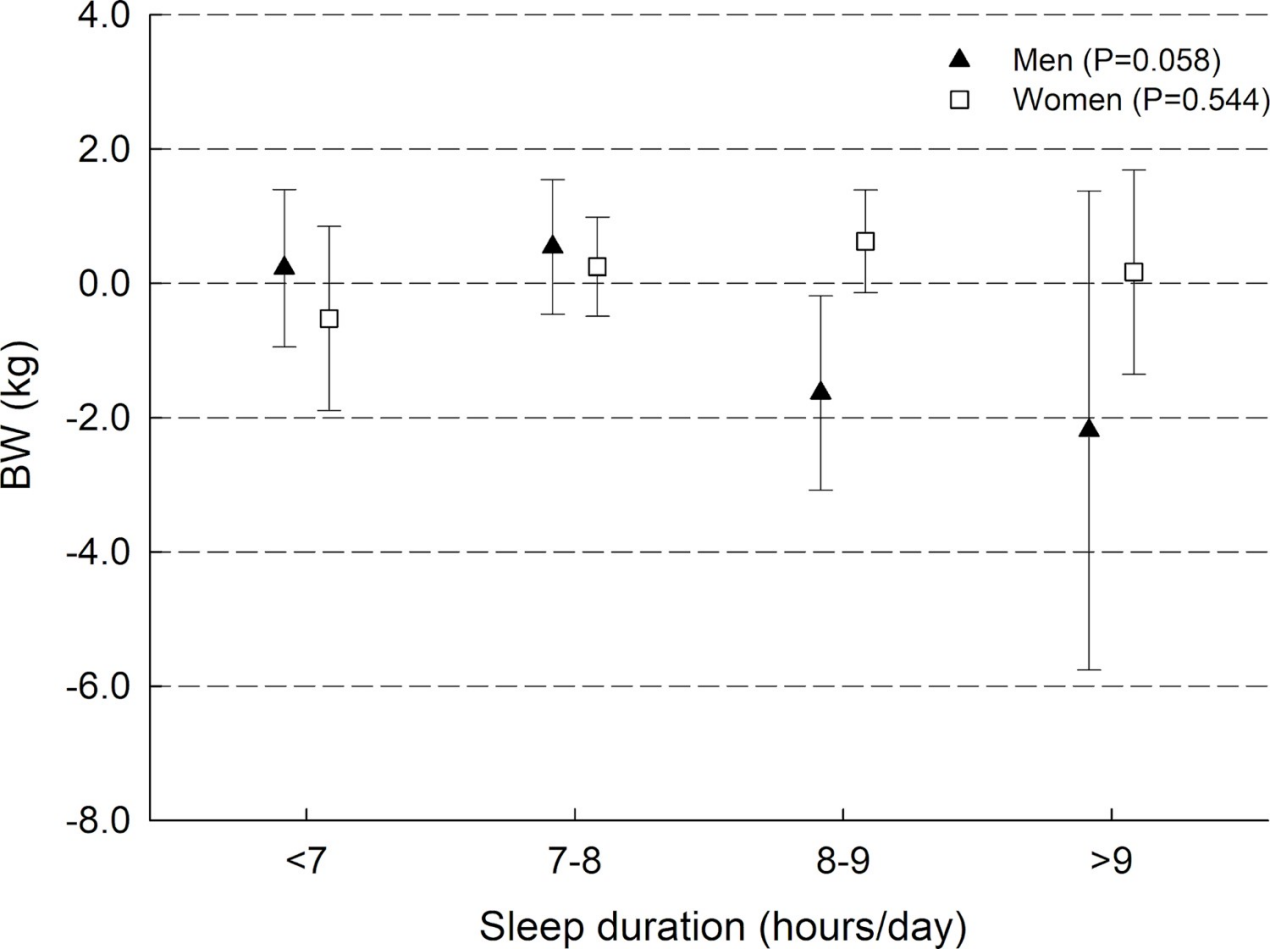
Mean values of 12-month changes in BW in categories of baseline sleep duration for men and women. Results presented as mean 12-month change (95% CI) in BW (ΔBW) in categories of sleep duration. Model with information on outcome, exposure, and baseline measure of outcome initial weight loss, physical activity, perceived stress, smoking status, frequency of alcohol consumption, education, sex, and age. Gender specific *P* values for differences in ΔBW according to categories of sleep duration were produced using analysis of covariance. BW, body weight.

We found some evidence of sex interaction in analysis of ΔBW (*P*-interaction = 0.037), but stratified analyses did not reveal statistically significant sex-specific associations **(Fig 1)**. We found no interaction between sleep duration and sex in analysis of ΔBF% (*P*-interaction = 0.834) and no interaction between sleep duration and intervention status in analyses of ΔBW (*P*-interaction = 0.160) or ΔBF% (*P*-interaction = 0.191).

Likewise, no association between baseline sleep onset and ΔBW or ΔBF% was observed. After adjusting for potential confounders and total sleep duration, we found an association between sleep onset and ΔDBP (*P* = 0.015), with the highest ΔDBP observed among participants with a sleep onset between 22:00 and 00:00 (0.20 mm Hg [95% CI −0.37 to 0.76]) and the lowest among participants with a sleep onset between 19:00 and 22:00 (−2.97 mm Hg [95% CI −4.99 to −0.94]). Moreover, we found an association between sleep onset and ΔTG (*P* = 0.028), with the highest ΔTG observed among participants with a sleep onset between 19:00 and 22:00 (0.80 mmol/L [95% CI 0.46 to 1.13]) and the lowest ΔTG among those with a sleep onset between 03:00 and 06:00 (0.25 mmol/L [95% CI −0.23 to 0.74]) **(Table 4).**

**Table 4:** Mean values of 12-month changes in BW, composition, and secondary outcomes in categories of baseline sleep onset.

Parameters	19:00–22:00 (*n* = 41)		22:00–00:00 (*n* = 548)		00:00–03:00 (*n* = 358)		03:00–06:00 (*n* = 20)		
	Mean^1^	(95% CI)	Mean	(95% CI)	Mean	(95% CI)	Mean	(95% CI)	*P*^5^
**ΔBW (kg)**									
Crude^2^	0.12	(−1.76 to 1.99)	0.09	(−0.42 to 0.61)	0.29	(−0.34 to 0.93)	0.09	(−2.63 to 2.81)	0.972
Adjusted^3^	0.18	(−1.69 to 2.05)	0.14	(−0.38 to 0.66)	0.24	(−0.42 to 0.89)	−0.15	(−2.89 to 2.56)	0.991
Adjusted + sleep duration^4^	0.23	(−1.67 to 2.14)	0.13	(−0.40 to 0.66)	0.21	(−0.46 to 0.89)	0.25	(−2.53 to 3.03)	0.997
**ΔBF%**									
Crude	−0.44	(−2.04 to 1.17)	−0.44	(−0.88 to 0.00)	−1.11	(−1.65 to −0.56)	−0.96	(−3.26 to 1.34)	0.312
Adjusted	−0.87	(−2.45 to 0.70)	−0.70	(−1.14 to −0.27)	−0.69	(−1.24 to −0.14)	−0.47	(−2.76 to 1.81)	0.994
Adjusted + sleep duration	−0.93	(−2.53 to 0.68)	−0.71	(−1.16 to −0.27)	−0.66	(−1.23 to −0.10)	−0.43	(−2.77 to 1.92)	0.987
**ΔSBP (mm Hg)**									
Crude	−3.27	(−6.27 to −0.27)	−0.72	(−1.54 to 0.10)	−2.12	(−3.13 to −1.10)	−1.12	(−5.42 to 3.18)	0.110
Adjusted	−3.85	(−6.84 to −0.87)	−0.82	(−1.65 to 0.01)	−1.85	(−2.89 to −0.80)	−1.94	(−6.26 to 2.37)	0.163
Adjusted + sleep duration	−3.95	(−6.98 to −0.91)	−0.87	(−1.71 to −0.02)	−1.80	(−2.87 to −0.72)	−1.40	(−5.82 to 3.02)	0.182
**ΔDBP (mm Hg)**									
Crude	−2.71	(−4.70 to −0.72)	0.14	(−0.41 to 0.68)	−0.52	(−1.19 to 0.16)	−0.32	(−3.17 to 2.53)	0.038
Adjusted	−2.99	(−4.98 to −1.00)	0.17	(−0.38 to 0.73)	−0.50	(−1.19 to 0.20)	−1.14	(−4.01 to 1.74)	0.017
Adjusted + sleep duration	−2.97	(−4.99 to −0.94)	0.20	(−0.37 to 0.76)	−0.54	(−1.26 to 0.18)	−1.10	(−4.06 to 1.85)	0.015
**ΔLDL (mmol/L)**									
Crude	−0.21	(−0.41 to 0.00)	−0.01	(−0.06 to 0.05)	−0.05	(−0.12 to 0.02)	−0.07	(−0.36 to 0.22)	0.299
Adjusted	−0.21	(−0.42 to −0.01)	0.01	(−0.05 to 0.06)	−0.07	(−0.14 to 0.00)	−0.12	(−0.42 to 0.17)	0.099
Adjusted + sleep duration	−0.21	(−0.42 to 0.00)	0.01	(−0.05 to 0.07)	−0.07	(−0.14 to 0.00)	−0.14	(−0.43 to 0.16)	0.098
**ΔHDL (mmol/L)**									
Crude	0.14	(0.05 to 0.23)	0.16	(0.14 to 0.19)	0.15	(0.12 to 0.18,	0.13	(0.00 to 0.26)	0.885
Adjusted	0.11	(0.03 to 0.20)	0.15	(0.13 to 0.18)	0.17	(0.14 to 0.20)	0.17	(0.05 to 0.30)	0.697
Adjusted + sleep duration	0.13	(0.04 to 0.22)	0.16	(0.13 to 0.18)	0.16	(0.13 to 0.20)	0.17	(0.04 to 0.30)	0.892
**ΔTG (mmol/L)**									
Crude	0.82	(0.50 to 1.15)	0.30	(0.21 to 0.38)	0.24	(0.13 to 0.34)	0.16	(−0.31 to 0.62)	0.009
Adjusted	0.81	(0.49 to 1.14)	0.28	(0.19 to 0.38)	0.25	(0.14 to 0.36)	0.21	(−0.26 to 0.68)	0.016
Adjusted + sleep duration	0.80	(0.46 to 1.13)	0.28	(0.19 to 0.37)	0.26	(0.14 to 0.37)	0.25	(−0.23 to 0.74)	0.028
**ΔHbA1c (%)**									
Crude	0.06	(−0.02 to 0.13)	0.08	(0.06 to 0.10)	0.08	(0.06 to 0.11)	0.13	(0.03 to 0.23)	0.667
Adjusted	0.04	(−0.03 to 0.12)	0.08	(0.06 to 0.10)	0.09	(0.06 to 0.11)	0.12	(0.01 to 0.22)	0.626
Adjusted + sleep duration	0.03	(−0.04 to 0.11)	0.07	(0.05 to 0.09)	0.09	(0.07 to 0.12)	0.12	(0.01 to 0.22)	0.411

^1^Results presented as mean 12-month change (95% CI) in categories of sleep onset.

^2^Model with information on outcome, exposure, and baseline measure of outcome.

^3^Adjusted for intervention status, initial weight loss, physical activity, perceived stress, smoking status, frequency of alcohol consumption, education, sex, and age.

^4^Same as adjusted + total sleep duration.

^5^*P* values for difference between sleep onset categories were produced using analysis of covariance.

Abbreviations: BF%, body fat percentage; BW, body weight; DBP, diastolic blood pressure; HbA1c, glycated haemoglobin; HDL, high-density lipoprotein; LDL low-density lipoprotein; SBP, systolic blood pressure; TG, triglyceride; Δ, 12-month change

In analyses of primary outcomes, we found no evidence of interaction between sleep onset and sex or interventions status (all *P*-interaction >0.061). Moreover, all analyses of primary outcomes remained statistically nonsignificant in analyses with sleep duration and sleep onset included as continuous variables **(S1 Table)** and after adjusting for number of sleep records (**S2 Table** and **S3 Table**).

### Variability in sleep duration and sleep onset

No associations between variability in sleep duration and subsequent change in ΔBW or ΔBF% were observed, but after adjusting for potential confounders and total sleep duration, we found that a higher between-day variability in sleep duration was associated with ΔHbA1c (0.02% per hour [95% CI 0.00 to 0.05]; *P* = 0.045). Moreover, in the fully adjusted models, we found that a higher between-day variability in time of sleep onset was associated with ΔBW (0.55 kg per hour [95% CI 0.10 to 0.99]; *P* = 0.016) and ΔBF% (0.41% per hour [95% CI 0.04 to 0.78]; *P* = 0.031). No associations between sleep onset variability and subsequent changes in secondary outcomes were observed **(Table 5).**

**Table 5:** Association between sleep duration and sleep onset variability and subsequent 12-month change in BW, composition, and secondary outcomes.

Parameters	Sleep duration			Sleep onset		
*N* = 967	β^1^	(95% CI)	*P*	β^1^	(95% CI)	*P*
**ΔBW (kg)**						
Crude^2^	0.17	(−0.37 to 0.71)	0.541	0.58	(0.14 to 1.02)	0.009
Adjusted^3^	0.11	(−0.42 to 0.65)	0.678	0.51	(0.07 to 0.95)	0.022
Adjusted + sleep duration^4^	0.27	(−0.31 to 0.84)	0.359	0.55	(0.10 to 0.99)	0.016
**ΔBF%**							
Crude	0.10	(−0.36 to 0.56)	0.668	0.33	(−0.04 to 0.71)	0.080
Adjusted	0.08	(−0.37 to 0.53)	0.726	0.40	(0.03 to 0.76)	0.035
Adjusted + sleep duration	0.10	(−0.38 to 0.58)	0.689	0.41	(0.04 to 0.78)	0.031
**ΔSBP (mm Hg)**						
Crude	0.06	(−0.81 to 0.92)	0.899	0.41	(−0.29 to 1.12)	0.247
Adjusted	0.15	(−0.71 to 1.01)	0.733	0.52	(−0.18 to 1.22)	0.146
Adjusted + sleep duration	0.18	(−0.74 to 1.10)	0.704	0.61	(−0.10 to 1.32)	0.092
**ΔDBP (mm Hg)**						
Crude	0.11	(−0.46 to 0.68)	0.705	0.17	(−0.30 to 0.63)	0.475
Adjusted	0.10	(−0.48 to 0.68)	0.732	0.16	(−0.31 to 0.63)	0.497
Adjusted + sleep duration	0.08	(−0.55 to 0.68)	0.829	0.14	(−0.33 to 0.62)	0.551
**ΔLDL (mmol/L)**						
Crude	0.00	(−0.06 to 0.06)	0.992	−0.02	(−0.06 to 0.03)	0.490
Adjusted	0.00	(−0.06 to 0.06)	0.992	−0.02	(−0.07 to 0.03)	0.377
Adjusted + sleep duration	−0.00	(−0.07 to 0.06)	0.921	−0.02	(−0.07 to 0.03)	0.362
**ΔHDL (mmol/L)**						
Crude	0.02	(−0.01 to 0.04)	0.231	−0.01	(−0.03 to 0.02)	0.619
Adjusted	0.01	(−0.01 to 0.04)	0.408	−0.00	(−0.02 to 0.02)	0.747
Adjusted + sleep duration	0.01	(−0.01 to 0.04)	0.371	−0.01	(−0.03 to 0.02)	0.608
**ΔTG (mmol/L)**						
Crude	−0.01	(−0.11 to 0.08)	0.796	0.05	(−0.03 to 0.12)	0.205
Adjusted	−0.01	(−0.10 to 0.09)	0.855	0.06	(−0.02 to 0.13)	0.141
Adjusted + sleep duration	−0.02	(−0.12 to 0.09)	0.752	0.06	(−0.01 to 0.14)	0.103
**ΔHbA1c (%)**						
Crude	0.02	(−0.00 to 0.04)	0.109	0.01	(−0.00 to 0.03)	0.114
Adjusted	0.02	(−0.00 to 0.04)	0.070	0.01	(−0.01 to 0.03)	0.186
Adjusted + sleep duration	0.02	(0.00 to 0.05)	0.045	0.01	(−0.00 to 0.03)	0.147

^1^Results presented as mean 12-month change in outcomes (95% CI) per additional hour of between-day variability in baseline sleep duration or sleep onset.

^2^Model with information on outcome, exposure, and baseline measure of outcome.

^3^Adjusted for intervention status, initial weight loss, physical activity, perceived stress, smoking status, frequency of alcohol consumption, education, sex, and age.

^4^Same as adjusted + total sleep duration.

Abbreviations: BF%, body fat percentage; BW, body weight; DBP, diastolic blood pressure; HbA1c, glycated haemoglobin; HDL, high-density lipoprotein; LDL low-density lipoprotein; SBP, systolic blood pressure; TG, triglyceride; Δ, 12-month change

Additional adjustment for the number of sleep records in analyses of primary outcomes gave essentially the same results, although the association between sleep onset variability and ΔBF% did not reach statistical significance (*P* = 0.085) (**S4 Table**). Finally, we found no evidence of interaction between sleep duration variability or sleep onset variability and sex or intervention status in any analyses of the primary outcomes (all *P*-interaction >0.175).

## Discussion

In a large WLM trial of European men and women who were overweight or had obesity and had achieved a clinically significant weight loss in the 12 months prior to inclusion at baseline, we found no evidence that sleep duration, sleep duration variability, or sleep onset was associated with WLM or ΔBF%. However, a higher sleep onset variability was associated with greater weight gain and increase in ΔBF%. Analyses of the secondary exploratory outcomes suggested that a higher between-day variability in sleep duration may be associated with an increase in ΔHbA1c, and that early sleep onset may be associated with a lower ΔDBP but a higher ΔTG.

We found few previous studies examining the relationship between sleep duration or quality and WLM. Ross and colleagues (2016) found that successful weight loss maintainers were more likely to report longer sleep duration compared to treatment-seeking adults with obesity [7]. Moreover, Yannakoulia and colleagues (2017) found that self-reported sleep quality was associated with WLM status [8]. However, as pointed out in both articles, their use of self-reported sleep data and cross-sectional designs make these previous results susceptible to report biases and reverse causality. While our prospective study, with objectively measured information on sleep duration and quality, could not confirm an association between baseline sleep duration and subsequent improved WLM, we found that a high sleep onset variability may predict subsequent weight regain. In line with this result, Patel and colleagues (2014) found that objectively measured sleep midpoint variability was associated with higher BMI in elderly men at a cross-sectional level [30]. Also, several studies have suggested that individuals with a high work-related variability in sleep onset, such as those involved in shift work, are more likely to develop overweight or obesity [31,32].

There are some potential explanations for a relationship between sleep onset variability and weight gain/regain. Although we found no previous studies examining this, it seems likely that individuals following a structured/consistent sleep schedule in general also have a higher degree of structure in other aspects of their lives, including factors directly or indirectly related to energy balance behaviour [33]. Moreover, several psychological factors (i.e., depression, mood disturbance, and stress) have been found to be associated with both irregular sleep patterns [34] and obesity [35]. Although psychological factors or different personality traits may to some degree explain the observed association, a biological link is also partly supported by the published literature. Greater sleep onset variability is associated with reduced perceived sleep quality [36], and reduced perceived sleep quality is associated with increased subjective appetite, metabolic changes in appetite hormones, increased energy intake, decreased basic metabolic rate, and decreased physical activity [37]. Moreover, studies in mice have suggested that circadian dysfunction, caused by genetic or environmental manipulation, may induce metabolic changes that lead to obesity [38–40]. An inconsistent sleep onset is also likely to cause eating irregularity, which has been linked to a lower thermic effect of foods [41] and a higher risk of obesity [42]. Additionally, although still poorly understood, it has also been suggested that both the central and peripheral circadian clock can be affected by a high-fat diet [43], and thus it is possible that both sleep onset irregularity and the associated weight gain are caused by dietary intake.

The data also show associations between elements of sleep and some of the secondary outcomes (e.g., that a higher between-day variability in sleep duration was associated with an increase in ΔHbA1c, and early sleep onset was associated with a lower ΔDBP but a higher ΔTG). While we found no studies directly confirming these associations, both low and high sleep duration have been linked to higher levels of HbA1c at a cross-sectional level [44], and prolonged sleep onset latency, insomnia, and shift work have been found to increase the odds of hypertension [45–47]. Likewise, we found no studies examining the association between time of sleep onset and ΔTG, but as intake of dietary fat has been found to be associated both with different elements of sleep [43,48] and ΔTG [49], this association may be caused by dietary intake. However, due to the exploratory nature of these analyses, we did not adjust for multiple testing, and thus these results should be interpreted with caution due to the risk of type I errors. Had Bonferroni adjustment been applied to account for the number of tests conducted, the results would not have been statistically significant.

The present study has several strengths, including the use of data from a large WLM trial and its prospective nature, which reduces the risk of reverse causality. Moreover, data on sleep were objectively measured during a 14-day period, providing us with a valid estimates of sleep duration, timing, as well as regularity of patterns across nights. A recent validation study conducted by de Zambotti and colleagues (2018) [22] concluded that the FC2 device showed promise in detecting sleep relative to polysomnography among 44 adults. The study reported a sensitivity of 0.96 (accuracy to detect sleep) and a specificity of 0.61 (accuracy to detect time awake). Although the FC2 significantly overestimated total sleep duration by 9 minutes compared to polysomnography, only 1 participant out of the 44 included participants fell outside the Bland–Altman limits of agreement for sleep duration [22]. Likewise, ΔBW, ΔBF%, and the obesity-related metabolic markers were objectively measured using validated instruments [19,24–26]. Finally, we had verified information on several lifestyle factors, including objectively measured physical activity, allowing us to adjust for potential confounding from these factors.

Our study also has some limitations. We used 14 days of sleep data recorded from day 3 to day 16 of the trial as a measure of baseline sleep. Because the trial had technically begun at that time, we cannot exclude that the intervention has affected the sleep variables. We can also not exclude that the results could be biased by the different strategies of WLM across the study population. However, as we found no evidence of intervention interactions, it seems unlikely that these factors had a substantial effect on the observed associations. We made comparison of ΔBW, ΔBF%, and secondary outcomes across individuals who vary in baseline sleep patterns. As people have different hereditary traits and are likely to vary according to other factors than their sleep habits, confounding is a potential problem, and although we adjusted our analyses for several baseline factors, it is still likely that some unmeasured or residual confounding remained. As an example, we did not adjust for sleep-disordered breathing, insomnia complaints, difficulty initiating sleep, meal timing, or eating behaviour. In addition, while we had information on objectively measured sleep and outcome measures from 967 individuals followed over a 12-month intervention period, we cannot exclude that we may have overlooked some associations as a result of insufficient statistical power. Relatedly, only 23 individuals had a daily sleep duration of <6 hours. Moreover, as only three individuals had a mean sleep onset between 06:00 and 19:00, these were excluded from the analyses, and consequently we could not confirm if sleep onset during this time interval was related to subsequent WLM.

Our results originate from a specific group of men and women with overweight or obesity who had successfully achieved a clinically significant weight loss prior to enrolment in the NoHoW trial. During the follow-up period they received a digital intervention based on self-regulation, motivation, and emotion regulation tools designed to improve WLM. Although we found no evidence of an interaction by intervention status, generalisation to the general population should be done with caution. Moreover, because our population consisted mainly of individuals with overweight and obesity, these results may not apply to individuals in the remaining BMI categories.

In conclusion, we found no evidence that the average duration or timing of sleep was related to WLM, but the results suggested that maintaining a consistent sleep onset may be associated with improved subsequent WLM. However, intervention studies are needed to determine whether this relationship is causal, a reflection of sleep quality or personality traits related to both sleep patterns and WLM. Sleep onset and variability in sleep duration may also be associated with subsequent change in different metabolic markers, but because of multiple testing, these exploratory outcomes should be interpreted cautiously.

## Supporting information

S1 STROBE Checklist Strobe, Strengthening the Reporting of Observational Studies in Epidemiology. (DOCX)

S1 Table Association between sleep duration or sleep onset (as continuous variables) and subsequent 12-month change in BW and BF%. BF%, body fat percentage; BW, body weight.
(DOCX)

S2 Table Mean values of 12-month changes in BW and composition in categories of baseline sleep duration, with additional adjustment for number of sleep records. BW, body weight
(DOCX)

S3 Table Mean values of 12-month changes in BW and composition in categories of baseline sleep onset, with additional adjustment for number of sleep records. BW, body weight
(DOCX)

S4 Table Association between sleep duration and sleep onset variability and subsequent 12-month change in BW and composition, with additional adjustment for number of sleep records. BW, body weight
(DOCX)

S1 Fig Flowchart illustrating the inclusion/exclusion of individuals in the NoHoW study. NoHoW, Navigating to a Healthy Weight
(DOCX)

S1 Text Publication proposal. (DOCX)

## Abbreviations

BF%: body fat percentage
BW: body weight
DBP: diastolic blood pressure
FC2: Fitbit Charge 2
HbA1c: glycated haemoglobin
HDL: high-density lipoprotein
ICT: information and communications technology
ISCED: International Standard Classification of Education
LDL: low-density lipoprotein
NoHoW: Navigating to a Healthy Weight
SBP: systolic blood pressure
STROBE: Strengthening the Reporting of Observational Studies in Epidemiology
TG: triglyceride
WLM: weight loss maintenance
