# Supplementary material for: Consistent sleep onset and maintenance of body weight after weight loss: An analysis of data from the NoHoW trial
Source: PLoS Med. 2020 Jul 16;17(7):e1003168. doi: 10.1371/journal.pmed.1003168 (PMC7365417; doi:10.1371/journal.pmed.1003168)
Supplement: S1 Table — BF%, body fat percentage; BW, body weight. (DOCX) [file pmed.1003168.s002.docx]

**S1 Table.** Association between sleep duration or sleep onset (as continuous variables) and subsequent 12-month change in body weight and body fat percentage

|  | **∆BW (n=967)** | | | **∆BF% (n=967)** | | |
| --- | --- | --- | --- | --- | --- | --- |
|  | **β** | **95% CI** | **P** | **β** | **95% CI** | **P** |
| **Sleep duration (hours/day)^1^** |  |  |  |  |  |  |
| Crude^2^ | -0.10 | -0.53, 0.32 | 0.637 | 0.27 | -0.09, 0.63 | 0.147 |
| Adjusted^3^ | -0.16 | -0.61, 0.30 | 0.504 | -0.04 | -0.43, 0.33 | 0.812 |
| **Sleep onset (hours from midnight)^4^** | | | | | | |
| Crude | 0.19 | -0.12, 0.51 | 0.234 | -0.23 | -0.50, 0.04 | 0.097 |
| Adjusted | 0.15 | -0.18, 0.48 | 0.380 | 0.05 | -0.23, 0.33 | 0.713 |
| Adjusted + sleep duration^5^ | 0.18 | -0.18, 0.54 | 0.325 | 0.08 | -0.23, 0.78 | 0.624 |

^1^Results presented as change in outcomes per hour of total sleep duration. ^2^Model with information on outcome, exposure and baseline measure of outcome. ^3^Adjusted for intervention status, initial weight loss, physical activity, perceived stress, smoking status, frequency of alcohol consumption, education, sex and age. ^4^Results presented as change in outcomes per hour of sleep onset relative to midnight. ^5^Same as adjusted + total sleep duration.
