# Supplementary material for: Consistent sleep onset and maintenance of body weight after weight loss: An analysis of data from the NoHoW trial
Source: PLoS Med. 2020 Jul 16;17(7):e1003168. doi: 10.1371/journal.pmed.1003168 (PMC7365417; doi:10.1371/journal.pmed.1003168)
Supplement: S3 Table — BW, body weight (DOCX) [file pmed.1003168.s004.docx]

**S3 Table.** Mean values of 12-month changes in body weight and composition in categories of baseline sleep onset with additional adjustment for number of sleep records.

|  | **19:00-22:00 (n=41)** | | **22:00-00:00 (n=548)** | | **00:00-03:00 (n=358)** | | **03:00-06:00 (n=20)** | |  |
| --- | --- | --- | --- | --- | --- | --- | --- | --- | --- |
|  | **Mean^1^** | **(95% CI)** | **Mean** | **(95% CI)** | **Mean** | **(95% CI)** | **Mean** | **(95% CI)** | **P^5^** |
| **ΔBW^2^ (kg)** |  |  |  |  |  |  |  |  |  |
| Adjusted^3^ | 0.18 | (-1.69, 2.05) | 0.17 | (-0.35, 0.69) | 0.19 | (-0.46, 0.85) | -0.32 | (-3.04, 2.41) | 0.988 |
| Adjusted + sleep duration^4^ | 0.22 | (-1.68, 2.12) | 0.17 | (-0.36, 0.70) | 0.17 | (-0.51, 0.84) | 0.15 | (-2.63, 2.93) | 0.999 |
| **ΔBF%** |  |  |  |  |  |  |  |  |  |
| Adjusted | -0.87 | (-2.45, 0.70) | -0.66 | (-1.10, -0.23) | -0.73 | (-1.28, -0.18) | -0.66 | (-2.94, 1.62) | 0.993 |
| Adjusted + sleep duration | -0.94 | (-2.53, 0.66) | -0.68 | (-1.12, -0.23) | -0.71 | (-1.28, -0.14) | -0.52 | (-2.86, 1.82) | 0.989 |

^1^ Results presented as mean 12-month change (95% CI) in categories of sleep onset. ^2^ Abbreviations: Body weight (BW), body fat percentage (BF%), and 12-month change (Δ). ^3^ Model with information on outcome, exposure, baseline measure of outcome, intervention status, initial weight loss, physical activity, perceived stress, smoking status, frequency of alcohol consumption, education, sex, age and number of records. ^4^ Same as adjusted + total sleep duration. ^5^ P-value for difference between sleep onset categories were produced using analysis of covariance.
