# Supplementary material for: Consistent sleep onset and maintenance of body weight after weight loss: An analysis of data from the NoHoW trial
Source: PLoS Med. 2020 Jul 16;17(7):e1003168. doi: 10.1371/journal.pmed.1003168 (PMC7365417; doi:10.1371/journal.pmed.1003168)
Supplement: S4 Table — BW, body weight (DOCX) [file pmed.1003168.s005.docx]

**S4 Table.** Association between sleep duration and sleep onset variability and subsequent 12-month change in body weight and composition with additional adjustment for number of sleep records.

|  | **Sleep duration** | | | **Sleep onset** | | | |  |
| --- | --- | --- | --- | --- | --- | --- | --- | --- |
| **N=967** | **β^1^** | **(95% CI)** | **P** | | **β^1^** | **(95% CI)** | **P** | |
| **ΔBW^2^ (kg)** | |  |  | |  |  |  | |
| Adjusted^4^ | 0.06 | (-0.48, 0.60) | 0.819 | | 0.45 | (0.03, 0.89) | 0.049 | |
| Adjusted + sleep duration^5^ | 0.21 | (-0.37, 0.79) | 0.473 | | 0.47 | (0.02, 0.92) | 0.040 | |
| **ΔBF%** |  |  |  | |  |  |  | |
| Adjusted | 0.02 | (-0.43, 0.48) | 0.916 | | 0.32 | (-0.05, 0.70) | 0.093 | |
| Adjusted + sleep duration | 0.04 | (-0.44, 0.53) | 0.867 | | 0.33 | (-0.05, 0.71) | 0.085 | |

^1^ Results presented as mean 12-month change in outcomes (95% CI) per additional hour of between day variability in baseline sleep duration or sleep onset. ^2^Abbreviations: Body weight (BW), body fat percentage (BF%), systolic blood pressure (SBP), diastolic blood pressure (DBP), low-density lipoproteins (LDL), high-density lipoprotein (HDL), glycated haemoglobin (HbA1c), and 12-month change (Δ). ^3^ Model with information on outcome, exposure and baseline measure of outcome. ^4^ Adjusted for intervention status, initial weight loss, physical activity, perceived stress, smoking status, frequency of alcohol consumption, education, sex and age. ^5^ Same as adjusted + total sleep duration.
