# Supplementary figures and images for: Consistent sleep onset and maintenance of body weight after weight loss: An analysis of data from the NoHoW trial
Source: PLoS Med. 2020 Jul 16;17(7):e1003168. doi: 10.1371/journal.pmed.1003168 (PMC7365417; doi:10.1371/journal.pmed.1003168)

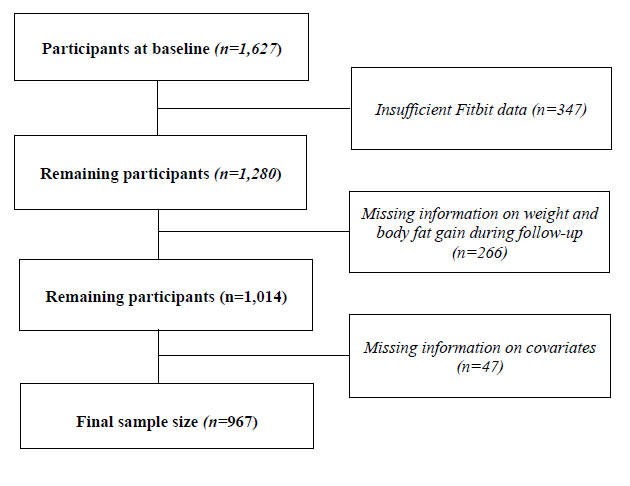


**S1 Fig.** Flowchart illustrating the inclusion/exclusion of individuals in the NoHoW study.

Supplement: S1 Fig — NoHoW, Navigating to a Healthy Weight (DOCX) [file pmed.1003168.s006.docx]
