# Supplementary material for: Consistent sleep onset and maintenance of body weight after weight loss: An analysis of data from the NoHoW trial
Source: PLoS Med. 2020 Jul 16;17(7):e1003168. doi: 10.1371/journal.pmed.1003168 (PMC7365417; doi:10.1371/journal.pmed.1003168)
Supplement: S1 Text — (DOCX) [file pmed.1003168.s007.docx]

**S1 Text.** Publication proposal (September 2018).

**Proposed lead author**

PhD-student (or Larsen SC).

**Proposed Co-Authors**

Heitmann BL, Stubbs J, Palmeira AL, Scott S, and others as agreed.

**Submission deadline**

Depends on data collection.

**Background**

Cross-sectional and longitudinal studies have suggested an association between insufficient sleep and obesity as well as weight gain (1, 2), but to our knowledge the relationship between sleep and weight loss maintenance has not been thoroughly explored. Thus, the aim of this study is to examine the association between sleep duration and quality with weight loss maintenance among the NoHoW participants.

**Hypotheses**

Short sleep duration and low sleep quality is directly associated with weight regain.

**Material and methods**

The study will consist of all NoHoW-participants with information on predefined variables.

*Measures*

- We will use Fitbit sleep information at baseline to estimate sleep duration and sleep quality (sleep duration variability, sleep onset and sleep onset variability).
- Baseline and follow-up information on adiposity measures
- Potential confounding factors.

*Statistical analyses*

Regression models adjusted for potential confounding factors, will be conducted to assess the associations of sleep duration and quality with change in outcomes. Gender and intervention interaction will be tested by adding product terms to the models and stratified analyzes will be conducted if appropriate.

**References**

1. Coughlin JW and Smith MT. Sleep, obesity, and weight loss in adults: is there a rationale for providing sleep interventions in the treatment of obesity? Int.Rev.Psychiatry 2014;26(2):177-188

2. Patel SR and Hu FB. Short sleep duration and weight gain: a systematic review. Obesity (Silver.Spring) 2008;16(3):643-653
